# Changes in expression of the chloride homeostasis-regulating genes, KCC2 and NKCC1, in the blood of cirrhotic patients with hepatic encephalopathy

**DOI:** 10.3892/etm.2012.721

**Published:** 2012-09-24

**Authors:** JUN-JIE LI, RU JI, YONG-QUAN SHI, YA-YUN WANG, YAN-LING YANG, KE-FENG DOU

**Affiliations:** 1 Department of Anatomy and K.K. Leung Brain Research Centre, Fourth Military Medical University;; 2 Departments of Hepatobiliary Surgery and; 3 Digestion, Xi-Jing Hospital, Fourth Military Medical University, Xi’an 710032, P.R. China

**Keywords:** chloride co-transporter, chloride homeostasis, hepatic encephalopathy

## Abstract

Hepatic encephalopathy (HE), a neuropsychiatric abnormality that commonly accompanies cirrhosis of the liver, is often difficult to treat and manage. Changes in chloride homeostasis are involved in the generation of a number of brain disorders. In this study, we considered whether chloride homeostasis is associated with HE. The mRNA levels of the Cl^−^ extrusion system (KCC2) and the Cl^−^ intrusion system (NKCC1) were detected by real-time RT-PCR in the plasma of 29 cirrhotic patients with HE of grade I-II, 36 cirrhotic patients with HE of grade III–IV, 20 cirrhotic patients without HE and 15 healthy controls. The mRNA levels of KCC2 in cirrhotic patients with mild and severe HE were significantly lower compared to those in cirrhotic patients without HE or in the healthy controls. However, NKCC1 mRNA levels did not differ between the different groups. In addition, for cirrhotic patients with HE, there were significant negative correlations between KCC2 levels and the levels of blood ammonia and hepatic function scores (Child-Pugh and model for end-stage liver disease scores); there was also a significant positive correlation between KCC2 levels and neurological status (Glasgow scores). In conclusion, our study indicates that an imbalance of KCC2 and NKCC1 may be a novel biomarker for detecting HE and for monitoring disease development.

## Introduction

Hepatic encephalopathy (HE) has been defined as a disturbance in central nervous system function due to hepatic insufficiency (1–3). Multiple treatments have been used for HE. However, their efficacy has been assessed infrequently by well-designed randomized clinical trials. This handicap reflects the difficulty in evaluating the wide range of neuropsychiatric symptoms. At the molecular level, HE is associated with a shift in the balance between inhibitory and excitatory neurotransmission towards a net increase in inhibitory neurotransmission, which is generally attributed either to an increased GABAergic tone or to alterations of the glutamatergic system (2,3). However, the nature of the respective control mechanisms remains to be completely resolved.

In the mammalian central nervous system, the intraneuronal chloride concentration ([Cl]i) determines the strength and polarity of GABA neurotransmission (4–6). The [Cl]i is to a large extent determined by the Na-K-2Cl co-transporter, NKCC1 (mediating Cl entry), and the K-Cl co-transporter, KCC2 (mediating Cl exit) (4,5). Changes in the expression of KCC2 and NKCC1 have been shown to be involved in the generation of brain disorders via the regulation of chloride homeostasis. NKCC1 activation has been reported to be involved in astrocyte swelling induced by ammonia and in brain edema in a thioacet-amide model of acute HE (6). However, the role of KCC2 in HE is unclear.

Therefore, the present study aimed to compare the plasma mRNA levels of KCC2 and NKCC1 in cirrhotic patients with grade III–IV HE with those in cirrhotic patients without HE, using the real-time RT-PCR technique. Additionally, we analyzed the associations between the mRNA levels of the two transporters and the corresponding hepatic functions, as well as the neurological status of cirrhotic patients with HE.

## Materials and methods

### Clinical subjects

Data from patients in the Departments of Hepatobiliary Surgery and Gastroenterology of Xi-Jing Hospital, Fourth Military Medical University, Xi’an, China, between June 2009 and December 2010 and from healthy controls, were collected and studied. A total of 85 liver disease patients, comprising 29 patients with HE grade I–II, 36 patients with HE grade III–IV, and 20 patients without HE, were evaluated in the present study. A total of 15 healthy controls were also included. Clinical characteristics of studied patients are presented in Table I. Written informed consent was obtained from the healthy volunteers and from each patient where this was possible. Clinical investigations were conducted according to the principles expressed in the Declaration of Helsinki. The study was approved by the Bioethical Committee of the Fourth Military Medical University.

A diagnosis of liver cirrhosis had already been made in all patients by pertinent clinical, laboratory and morphological procedures performed during previous hospitalization. Diagnosis and the grade of HE were assessed according to a detailed physical, neurological and psychometric assessment, with particular note being made of the mental status, the severity of asterixis and the performance in the number connection test type A. A complete neurological examination had been performed on every enrolled patient. To obtain objective clinical criteria for evaluating the clinical improvement of HE, we excluded patients with alcoholic liver cirrhosis to avoid bias by neurological and psychiatric signs due to chronic or acute ethanol abuse.

### Estimation of liver insufficiency and the neurological status of the subjects

To estimate a possible association between levels of mRNAs of the two transporters, KCC2 and NKCC1, on the one hand, and a corresponding degree of liver insufficiency and the neurological status of the individuals with liver cirrhosis on the other, Child-Pugh scores, model for end-stage liver disease (MELD) scales and Glasgow coma scores were evaluated in all included patients. Moreover, the MELD score, based on serum creatinine, albumin and bilirubin concentrations, was analyzed. Assessment of the neurological status of patients was performed according to the Glasgow coma score (7). The Glasgow score was recorded in each patient immediately prior to serum collection by a physician who was unaware of the experimental processes. Mid-upper arm muscle circumference (MAMC) and triceps skinfold thickness (TSF) were measured as indices of body fat and muscle protein compartment, respectively, using standard techniques, as previously reported. MAMC % (percentage of standard values) and TSF % (percentage of standard values) were considered to define the nutritional status. Glomerular filtration rate (GFR, as calculated from cystatin C) was measured as an index of renal function (8). Clinical characteristics of the studied population are presented in Table I. It should be indicated that the patients in groups A and B were under treatment with lactulose and diuretics.

Plasma KCC2 and NKCC1 levels were also compared with those in the 15 healthy volunteers (5 females and 10 males with a median age of 48 years, range 30–75 years).

### Real-time RT-PCR detection

Total RNA was extracted from serum as described by Vendittelli *et al* (9). The quality and integrity of total RNA was evaluated using gel electrophoresis and spectrophotometric determination. All samples were treated with RNase-free DNase I (10 U/l) (Boehringer Mannheim, Mannheim, Germany) and subsequently extracted with phenol/chloroform/isoamylic alcohol. Total RNA was reverse-transcribed by MMLV reverse transcriptase (Gibco BRL Life Technologies, Ltd., Paisley, UK) in the presence of an RNase inhibitor (Gibco BRL). For a quantitative comparison of mRNA levels, real-time PCR was performed using SYBR-Green fluorescence in a LightCycler^®^ System (Roche Diagnostic GmbH, Basel, Switzerland). The PCR reaction mixture was prepared using the SYBR-Green PCR Master Mix. Thermal cycling conditions were 10 min at 95°C followed by 35 cycles of 95°C for 15 sec and at 60°C for 1 min on thermal cycles (DFC-3200, MJ Research Company, Waltham, MA, USA). Amplification specificity was checked using melting curves. Both negative and positive controls were included in each PCR reaction. All assays were carried out three times as independent PCR runs for each cDNA sample. Gene expression was always related to the expression of GAPDH as the housekeeping gene, which is known to be a good choice for normalization of expression levels (10). Each gene expression was normalized with respect to GAPDH mRNA content. The sequences of the human primer for SYBR-Green PCR were as follows: KCC2 (145 bp, nucleotides 800–817, NM_020708 file in GenBank): GAAGTGCTCAGAGAGGTGG (sense) and GCAGAAGAG GAAGAAGGC (antisense); NKCC1 (140 bp, nucleotides 500–523, NM_001046 file in GenBank): AGGAGCATTCAA GCACAGCTAACA (sense) and CGCTCTGATGATTCC CACGA (antisense); GAPDH (147 bp): ACTTCAACAGCG ACACCCACT (sense) and GCCAAATTCGTTGTCATA CCAG (antisense). Calculations of expression were performed with the 2^ΔΔCT^ method according to a previous report (11). All measurements were carried out in the absence of information as regards the origin of the samples.

### Measurement of blood ammonia

To express the association between blood ammonia levels and KCC2/NKCC1 mRNA expression, the levels of ammonia in the blood from enrolled subjects with or without HE were measured.

For the determination of plasma ammonia levels, approximately 10 ml of blood was collected from each patient from a stasis-free vein in an EDTA container and stored in an ice bath. Adequate precautions were taken to avoid hemolysis, which interferes with the assay. The plasma was then separated and an ammonia assay was carried out within 20 min using a commercially available Randox kit (Jiancheng Laboratories Ltd., Nanjing, China). The corresponding increase in absorbance at 340 nm is proportional to the plasma ammonia concentration.

### Statistical analysis

All statistical analyses were performed using the statistical software package, SSPS 13.0 (SPSS Inc., Chicago, IL, USA). Data within 95% CI were used for analysis. Results are expressed as the means ± standard error of the mean (SE). The significance of differences was calculated by the non-parametric Mann-Whitney U test. For the correlation analysis, the Spearman non-parametric correlation was used. A value of P<0.05 was considered to indicate a statistically significant difference.

## Results

### Comparison of KCC2 and NKCC1 mRNA levels in peripheral blood of patients and control subjects

**Figure 1:**
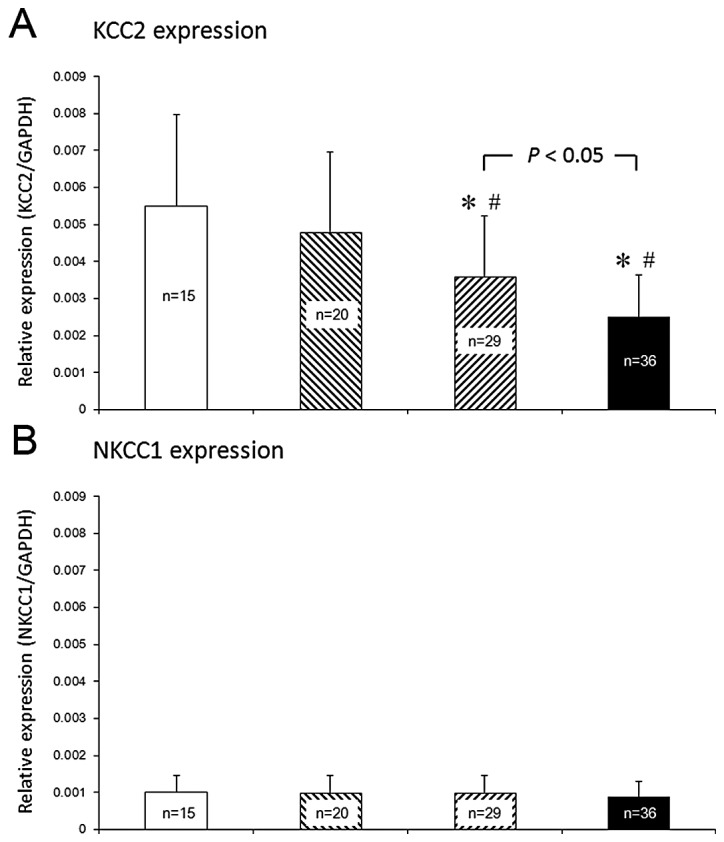
Relative mRNA expression levels of plasma (A) KCC2 and (B) NKCC1 in cirrhotic patients with grade I–II hepatic encephalopathy (HE), grade III–IV HE, when compared with cirrhotic patients without HE and healthy individuals. Data are presented as the means ± SE, ^*^P<0.05 vs. healthy controls and ^#^P<0.05 vs. cirrhotic patients without HE.

The means ± SEM of the relative KCC2 mRNA levels of the cirrhotic patients with grade I–II HE (0.0034±0.0011; n=29) were significantly lower (P<0.05, Fig. 1) than those of the cirrhotic patients without HE (0.0045±0.0023; n=20) or those of the healthy individuals (0.0055±0.0023; n=15). Moreover, the relative mRNA levels of KCC2 of the cirrhotic patients with grade III–IV HE (0.0021±0.0014; n=36) were much lower (P<0.05, Fig. 1) than those of patients with grade I–II HE.

The relative mRNA expression levels of KCC2/GAPDH did not show any significant association with gender (P=0.525), age (P=0.711), or liver cirrhosis etiology (P=0.114).

In contrast to this, the NKCC1 plasma concentrations in the cirrhotic patients with HE (0.0011±0.0007, n=36), in the cirrhotic patients without HE (0.0009±0.0007, n=20) and in the healthy controls (0.0010±0.0009, n=15) were all similar (Fig. 1B).

In addition, the relative mRNA expression levels of NKCC1/GAPDH in group A did not show any significant association with gender (P=0.462), age (P=0.369), or liver cirrhosis etiology (P=0.128).

### Association of KCC2 and NKCC1 mRNA levels with patient ammonia levels

As shown in Table I, all cirrhotic patients with HE (grades I–II and III–IV) showed higher ammonia levels than the upper limit of the range for the cirrhotic patients without HE (83.9 *μ*mol/l) or the healthy individuals (57.0 *μ*mol/l). Furthermore, the ammonia levels of patients with severe HE were higher than those of patients with mild HE.

**Figure 2:**
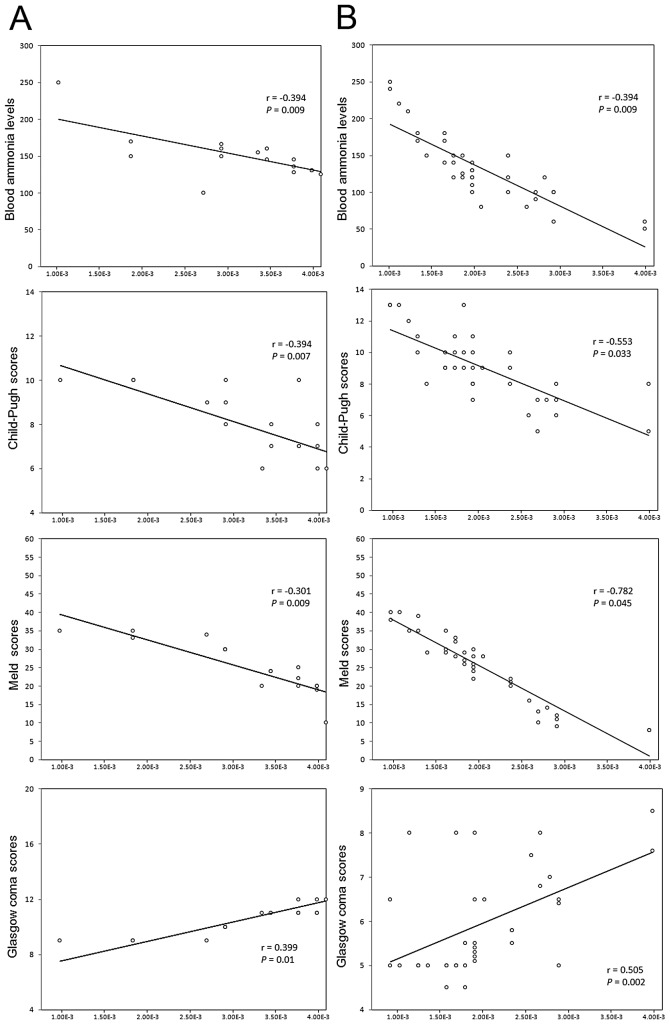
Correlation between the relative mRNA expression levels of plasma KCC2 with levels of blood ammonia, Child-Pugh scores, MELD scores and Glasgow coma scores in cirrhotic patients with (A) grade I–II hepatic encephalopathy (HE) and (B) grade III–IV HE.

As shown in Fig. 2, plasma KCC2 mRNA levels in all cirrhotic patients with HE showed a significant negative correlation with the levels of blood ammonia. Moreover, the KCC2 levels in patients with grade III–IV HE (Fig. 2B) were found to have a stronger correlation with ammonia levels than those in patients with grade I–II HE (Fig. 2A). Similarly, plasma KCC2 mRNA levels in cirrhotic patients without HE showed a significant negative correlation with the levels of blood ammonia (r=−0.388, P=0.002).

In contrast to this, the relative mRNA expression levels of NKCC1/GAPDH in all cirrhotic patients with HE or in cirrhotic patients without HE showed no significant association with the levels of blood ammonia (P=0.252 and 0.176, respectively).

### Association of KCC2 and NKCC1 mRNA levels with patient hepatic functions

As shown in Fig. 2, plasma KCC2 mRNA levels in all cirrhotic patients with HE were decreased in relation to the degree of liver insufficiency. This was demonstrated through a significant negative correlation with Child-Pugh scores and MELD scores. Moreover, the KCC2 levels in patients with grade III–IV HE (Fig. 2B) were found to have a stronger correlation with Child-Pugh and MELD scores than those in patients with grade I–II HE (Fig. 2A).

In contrast to this, the relative levels of NKCC1/GAPDH in the patients without HE showed no significant association with the Child-Pugh scores (P= 0.556) or the MELD scales (P= 0.188).

### Association of KCC2 and NKCC1 mRNA levels with patient neurological status

Plasma KCC2 levels showed a significant positive association with the Glasgow coma scores of patients who presented with HE (Fig. 2). Patients with grade III–IV HE (Fig. 2B) were found to have a stronger correlation with the Glasgow coma scores than those with grade I–II HE (Fig. 2A). In contrast to this, the relative mRNA expression levels of NKCC1/GAPDH in all cirrhotic patients with HE showed no significant association with Glasgow coma scores (P=0.354).

## Discussion

The main findings obtained in the present study in cirrhotic patients with HE are the decrease in the mRNA levels of KCC2 in the blood, indicating that an impaired GABAergic inhibition may be present in HE, in contradiction to the established hypothesis that attributes the pathophysiology of HE to increased GABAergic tone (12). Altered GABAergic neurotransmission was first implicated in the pathophysiology of HE based on the phenomenon of increased GABAergic tone in a rabbit model of fulminant hepatic failure (13). However, it should be noted that the evidence gathered since then is not unequivocal. GABA concentrations were found to be unaltered (14,15) or increased (13). Likewise, GABA_A_ receptor densities have been reported to be upregulated (16,17) in certain studies; however, the mRNA expression of the GABA transporter, GAT-2, has been shown to be increased in the cerebral cortex of the portacaval-shunted rat, whereas the genes for the GABA_B1D_ receptor and for the β2 subunit of the GABA_A_ receptor have been shown to be downregulated (18). Reports concerning GABA_A_-associated benzodiazepine (BZ) binding sites are also controversial. Additionally, attention should be paid to the fact that the majority of experimental approaches have made use of animal models (13–18), and only few studies were based on *in vivo* investigations of HE patients (19,20). The present results are based on comparisons of the mRNA levels of KCC2 and NKCC1 in the plasma of cirrhotic patients with severe HE (grade III–IV) and those without HE. This is the first report using blood samples from encephalopathy patients, thus reflecting the true nature of HE. Furthermore, our present results are consistent with the previous finding that there is a significant decrease in the GABAergic tone in the cerebral cortex of hyperammonemic rats (21). However, we should not focus on a single neurotransmitter system, which cannot represent the multifactorial approach required to elucidate the molecular mechanisms involved in the disease of HE. That is, the balance and imbalance between excitatory/inhibitory neurotransmissions may be more important than the change of any one variable.

Although the cause may be explained by a multifactorial theory, the accumulation of ammonia has been considered to play an important role in the pathogenesis of HE (1–3). The present results primarily indicate a possible association between decreased plasma mRNA levels of KCC2 and increased levels of blood ammonia, the degree of liver insufficiency, and the deteriorated neurological status of the patients. The expression of NKCC1 did not change, as is consistent with results obtained from thioacetamide-induced HE rats (22). The present results indicate that altered chloride homeostasis was closely related to the severity of the HE and may be a feature of neurological alterations present in HE. The imbalance of chloride homeostasis in HE patients suggested in this study is consistent with previous reports of changes in Cl^−^ transporters in an animal model of HE *in vivo* (21) and a culture model of hyperammonemia *in vitro* (6). Accumulating evidence suggests that abnormal chloride homeostasis, induced by the downregulation of KCC2, the upregulation of NKCC1, or both, is associated with neuronal trauma or brain disorders, such as epilepsy (23), neuropathic pain (24), or amyotrophic lateral sclerosis (25). It is known that diagnostic methods of modern medicine allow the recognition of functional abnormalities before the appearance of symptoms. Early recognition of impairment may allow the avoidance or delay of a disease. For this reason, the detection of an abnormal balance of chloride homeostasis or of KCC2/NKCC1 expression levels may be important for the early diagnosis of HE, and an early identification of patients at the initial phases of HE may improve the quality of life and the prognosis for these patients. Further studies are required to fully elucidate the mechanism and functional significance underlying the balance of KCC2 and NKCC1 in the manifestation of HE.

## Figures and Tables

**Table I:** Clinical characteristics of the studied patients.

	Liver cirrhosis		
Characteristic	without HE	with grade I–II HE	with grade III–IV HE
Age, years; median (range)	56 (36–77)	55 (33–76)	52 (34–74)
Gender, male/female	13/7	18/11	21/15
Liver cirrhosis etiology (n)			
PBC	1	6	5
HBV	19	22	25
HCV	0	1	6
GFR, ml/min/1.73 m^2^; range	52–90	35–90	35–90
Child-Pugh score, median (range)	6 (5–9)	6 (5–10)	8 (5–13)
MELD score, median (range)	17 (6–35)	20 (8–35)	26 (8–40)
Ascites (n)	12	13	28
ALT, U/l; mean ± SE	150.0±36.2	190.1±40.3	231.5±44.5
Bilirubin, mg/dl; mean ± SE	2.04±1.0	2.33±1.2	2.88±0.8
Albumins, g/dl; mean ± SE	4.11±1.1	3.25±1.3	2.95±0.9
Glasgow coma score, median (range)	12 (9.0–15.0)	11 (9.0–15.0)	6 (4.3–8.5)
Ammonia, *μ*mol/l; mean ± SE	50.2±33.7	134.4±40.2	210.1±79.0
MAMC % of standard, mean ± SE	97.0±20.6	90.0±18.7	88.5±15.6
TSF % of standard, mean ± SE	96.3±41.8	85.1±40.5	79.2±43.4

HE, hepatic encephalopathy; PBC, primary biliary cirrhosis; HBV, hepatitis B virus; HCV, hepatitis C virus; GFR, glomerular filtration rate; MELD, model for end-stage liver disease; ALT, alanine aminotransferase; SE, standard error; MAMC, mid-upper arm muscle circumference; TSF, triceps skinfold thickness.
